# Multiobjective Image Color Quantization Algorithm Based on Self-Adaptive Hybrid Differential Evolution

**DOI:** 10.1155/2016/2450431

**Published:** 2016-09-25

**Authors:** Zhongbo Hu, Qinghua Su, Xuewen Xia

**Affiliations:** ^1^School of Information and Mathematics, Yangtze University, Jingzhou, Hubei 434023, China; ^2^School of Software, East China Jiaotong University, Nanchang 330013, China

## Abstract

In recent years, some researchers considered image color quantization as a single-objective problem and applied heuristic algorithms to solve it. This paper establishes a multiobjective image color quantization model with intracluster distance and intercluster separation as its objectives. Inspired by a multipopulation idea, a multiobjective image color quantization algorithm based on self-adaptive hybrid differential evolution (MoDE-CIQ) is then proposed to solve this model. Two numerical experiments on four common test images are conducted to analyze the effectiveness and competitiveness of the multiobjective model and the proposed algorithm.

## 1. Introduction

Image color quantization is one of the common image processing techniques. It is the process of reducing the number of colors presented in a color image with less distortion [1]. Most of the image color quantization methods [2–12] are essentially based on data clustering algorithms. Recently, some heuristic methods, such as genetic algorithm (GA) [13, 14], particle swarm optimization algorithm (PSO) [15–17], and differential evolution (DE) [18–21], have been employed to solve the image color quantization problems which are considered as optimization problems. Evaluation criteria, which are used as objective functions of optimization problems, often incorporate mean square-error (MSE) [22–24], intracluster distance (${\bar{d}}_{max}$), and intercluster separation (*d*_min_) [25–28].

Most of the image color quantization algorithms based on heuristic methods are single-objective methods; that is, only one evaluation criterion is used. References [26–28] have used three evaluation criteria, but their three criteria have been merged to get a linear weighting objective function. In general, the objective function in any of the above algorithms holds only one evaluation criterion or a linear combination of several evaluation criteria. This paper presents the following two aspects:

- Develop multiobjective model for image color quantization problems. Based on the model, we can obtain a quantized image with the smallest color distortion among those images which meet a trade-off between the optimal color gradation and the optimal color details.
- Propose a multiobjective algorithm based on a self-adaptive DE for solving the multiobjective image color quantization model.

The rest of the paper is organized as follows.  Section 2 establishes a multiobjective image color quantization model.  Section 3 presents a multiobjective image color quantization algorithm based on self-adaptive hybrid DE (MoDE-CIQ). Experimental results and discussion on four test images are provided in Section 4. Conclusions are given in Section 5.

## 2. Establishment of a Multiobjective Image Color Quantization Model

### 2.1. Multiobjective Image Color Quantization Model

In single-objective models, mean square-error (MSE) (1) is the most popular evaluation criterion for color image quantization [29]. Intracluster distance (${\bar{d}}_{max}$) (2) and intercluster separation (*d*_min_) (3) come next in importance to MSE. Smaller MSE means smaller color distortion. Smaller ${\bar{d}}_{max}$ means smoother gradation of similar colors. Larger *d*_min_ means more color details to be preserved. The three evaluation criteria are expressed in the following formulas [28]:

(1) MSE=1M×N∑i=1M ∑j=1Nmink∈1,2,…,K⁡dIi,j,ck,

(2) d¯max=maxk=1,2,…,K⁡∑∀Ip∈CkdIp,ckCk,

(3) dmin=min∀k1,k2=1,2,…,K, k1≠k2⁡dck1,ck2.

Here, *M* × *N* is the size of a color image *I*. *I*(·, ·) is a pixel in *I*. *K* is a given color number of a colormap. *k* is the sequence number of the colors in the colormap. *c*_*k*_ is the *k*th color of the colormap. *k*_1_ and *k*_2_ are two different sequence numbers of the colors in the colormap.   *C*_*k*_ is the cluster of all pixels in *I* with similar color to *c*_*k*_. |*C*_*k*_| is the number of all pixels in *C*_*k*_. *I*_*p*_ is the color of a pixel in *C*_*k*_. *d*(·, ·) represents Euclidean distance.

This paper proposes a multiobjective image color quantization model which uses two evaluation criteria, ${\bar{d}}_{max}$ and *d*_min_, as its subobjective functions. The model can be formulized as follows:

(4) minimize Fx=g1x,g2xTs.t. x∈0,2553×K.

Here [0,255]^3×*K*^ is decision space. Decision vector *x* is a colormap consisting of *K* randomly selected color triples in the color space  [0,255]^3^. Let

(5) $$\begin{array}{c} c_{k}=\left(\;{x_{3k-2},x_{3k-1},x}_{3k}\;\right),\;k=1,2,\ldots ,K, \end{array}$$

be the *k*th color of the colormap. Then

(6) xc1,c2,…,cK=x1,x2,x3,x4,x5,x6,…,x3K−2,x3K−1,x3K.

*F*(*x*) is the objective function with the following two subobjectives:

(7) g1x=d¯max,g2x=255−dmin.

This model aims to make a trade-off between ${\bar{d}}_{max}$ minimum and *d*_min_ maximum. The solution set of this multiobjective model is called Pareto set, the solutions of which could balance color gradation and color details.

Obviously, the solution with the smallest MSE in the Pareto set of the above multiobjective model corresponds to a quantized image, which holds the smallest color distortion among those images with a balance between the optimal color gradation and the optimal color details.

### 2.2. Conflict Detection of the Subobjective Functions

As we all know, the subobjective functions of a multiobjective model should be conflicting. This means, as two subobjectives in the above model, *g*_1_(*x*) and *g*_2_(*x*) should not become better simultaneously. Namely, when ${\bar{d}}_{max}$ becomes better (smaller), *d*_min_ should not also become better (larger). In this part, several experiments are conducted to show that the subobjective functions, *g*_1_(*x*) and *g*_2_(*x*), in the above model are obviously conflicting.

**Figure 1:**
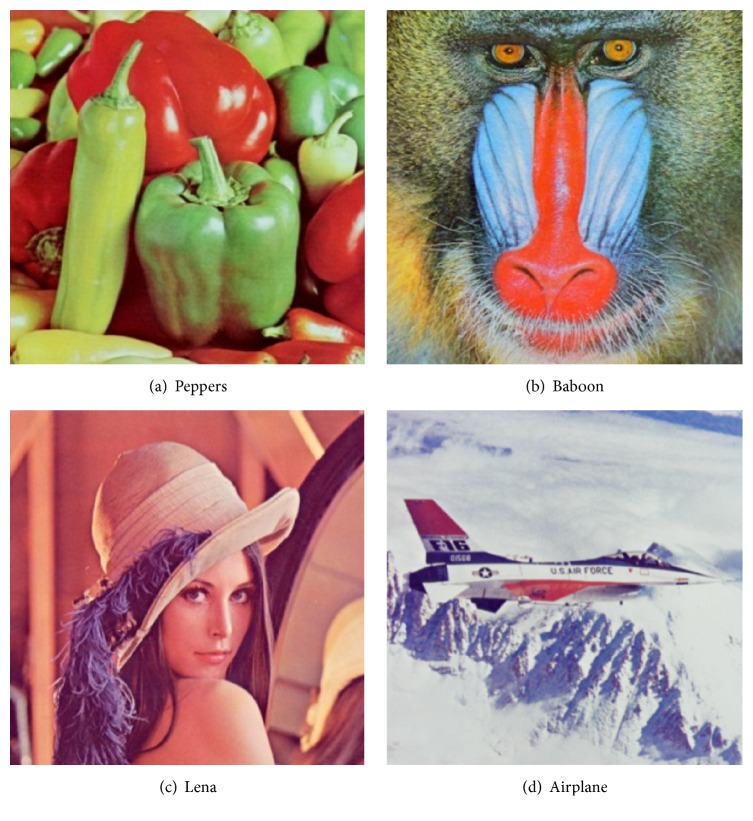
Test images.

Figure 1 shows four common test images (Peppers, Baboon, Lena, and Airplane) with size 512 × 512 pixels. Reference [15] presented a color image quantization algorithm based on self-adaptive hybrid DE (SaDE-CIQ), in which the objective function is MSE. We, respectively, replace its objective with ${\bar{d}}_{max}$ and *d*_min_ to obtain two algorithms, named SaDE-CIQ1 and SaDE-CIQ2. SaDE-CIQ, SaDE-CIQ1, and SaDE-CIQ2 are implemented to quantize all test images into the quantized images with 16 colors. Each algorithm is run 10 times on each test image. In the three algorithms, there are two parameters, a maximum iteration *t*_max_ and a mixed probability *p*. Here, *t*_max_ = 200. For showing the same relation of MSE, ${\bar{d}}_{max}$ and *d*_min_ for the different values of *p*, we let *p* take three different values, 0.1, 0.05, and 0.01 in the three algorithms.

**Figure 2:**
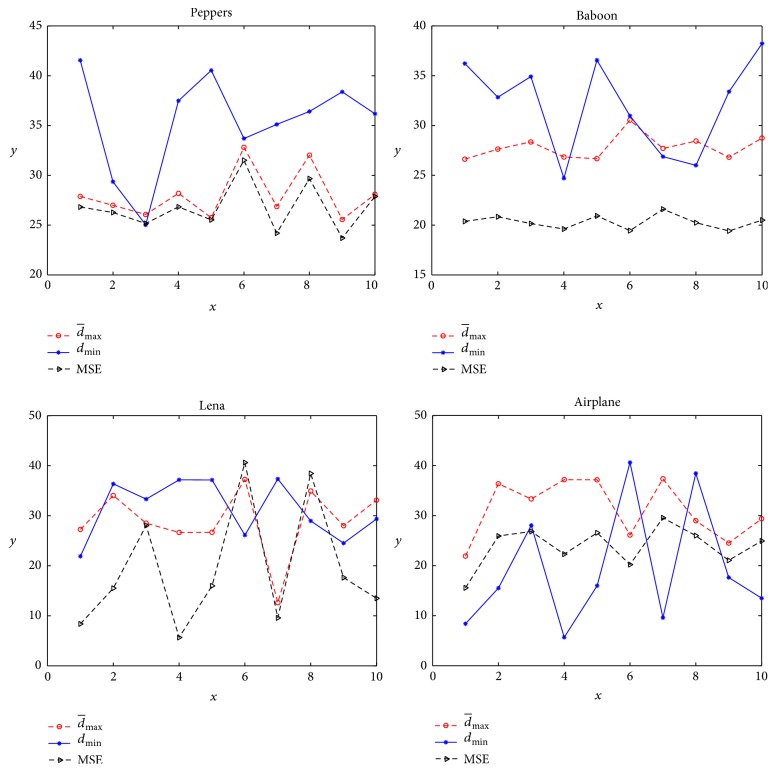
The curves of ${\bar{d}}_{max}$, *d*_min_, and MSE obtained by SaDE-CIQ1 (*p* = 0.1).

For the three algorithms with different *p*, we can get the similar relation of MSE, ${\bar{d}}_{max}$ and *d*_min_. So, we only use the part results of SaDE-CIQ1 with *p* = 0.1 as an example to analyze the relation of MSE, ${\bar{d}}_{max}$ and *d*_min_. By any image and its quantized image, we can calculate the values of MSE, ${\bar{d}}_{max}$ and *d*_min_.  Table 1 gives all the objective values ${\bar{d}}_{max}$ of SaDE-CIQ1 in 10 runs and the corresponding values of MSE and *d*_min_.  Figure 2 shows the changes of these values in 10 runs. We include the curves of Peppers from first run to second run as an example of how to illustrate the conflicts of MSE, ${\bar{d}}_{max}$ and *d*_min_. When ${\bar{d}}_{max}$ becomes better (smaller), *d*_min_ does not become better (larger). When MSE becomes better (smaller), *d*_min_ does not become better (larger). When ${\bar{d}}_{max}$ becomes better (smaller), MSE also becomes better (smaller). These mean ${\bar{d}}_{max}$ and *d*_min_ are conflicting, MSE and *d*_min_ are conflicting, and ${\bar{d}}_{max}$ and MSE are not conflicting. According to the statistical analysis for all test images, there are 15 conflicts between ${\bar{d}}_{max}$ and *d*_min_, 16 between MSE and *d*_min_, and 11 between ${\bar{d}}_{max}$ and MSE. These statistical data show that any two of MSE, ${\bar{d}}_{max}$ and *d*_min_, are in conflict.

In summary, for the conflict of ${\bar{d}}_{max}$ and *d*_min_, it is appropriate to select them as the subobjective functions in the above multiobjective image color model. Meanwhile, for the conflicts of MSE with ${\bar{d}}_{max}$ and *d*_min_, there does not exist preference when MSE is applied to select the solution in the Pareto set of the above multiobjective model.

## 3. Multiobjective Image Color Quantization Algorithm Based on Self-Adaptive Hybrid DE

For solving the above multiobjective image color quantization model, this section proposes a multiobjective image color quantization algorithm based on self-adaptive hybrid DE (MoDE-CIQ). This algorithm merges the ideas of SaDE-CIQ in [19] and a multipopulation DE algorithm VEDE [30], which is a Pareto-based multiobjective DE algorithm. The main steps of the proposed MoDE-CIQ algorithm are described as below.

**Step 1 (initialize populations).**

Two initial populations including NP individuals are randomly selected separately. Here, each individual is a colormap with *K* colors from an image *I*. The initial populations are denoted by

(8) X1=x1,x2,…,xNP,X2=xNP+1,xNP+2,…,x2∗NP.

**Step 2 (optimize populations).**

The population *X*_1_ is updated by SaDE-CIQ with *g*_1_(*x*) as its objective. The population *X*_2_ is updated by SaDE-CIQ with *g*_2_(*x*) as its objective. Then, the best individuals of the two populations are exchanged. The update and exchange operations are repeated to achieve a predetermined iteration number *t*_max_. The set of *t*_max_th generation individuals of the two populations is denoted by

(9) $$\begin{array}{c} X=\left\{\;x_{1}^{t_{max}},x_{2}^{t_{max}},\ldots ,x_{NP}^{t_{max}},x_{NP+1}^{t_{max}},x_{NP+2}^{t_{max}},\ldots ,x_{2*NP}^{t_{max}}\;\right\}. \end{array}$$

**Step 3 (reserve nondominated solutions).**

All nondominated solutions in *X* are recorded in a set POS.

(Note: for an individual *x*_*i*_^*t*_max_^  (*i* = 1, 2,…, 2*∗*NP), if there is no another one *x*_*j*_^*t*_max_^  (*j* ≠ *i*, *j* = 1, 2,…, 2*∗*NP) such that *g*_1_(*x*_*j*_^*t*_max_^) < *g*_1_(*x*_*i*_^*t*_max_^) and *g*_2_(  *x*_*j*_^*t*_max_^) < *g*_2_(*x*_*i*_^*t*_max_^), that is, *F*(*x*_*j*_^*t*_max_^)≺*F*(*x*_*i*_^*t*_max_^), it is a nondominated solution. Otherwise, it is a dominated solution.)

**Step 4 (obtain an approximative Pareto solution set).**

Steps 2 and 3 are repeated to achieve a predetermined iteration number* Loop*. The final set POS is recorded as an approximative Pareto solution set.

**Step 5 (determine an optimal solution).**

In the set POS, the solution with the smallest values of MSE is finally reserved as an optimal solution of an image color quantization problem.

The pseudocode of MoDE-CIQ is shown as Pseudocode 1.

## 4. Numerical Experiments

In this section, two sets of experiments are conducted to illustrate the effectiveness of MoDE-CIQ algorithm and the advantage of the multiobjective model, respectively.

### 4.1. Experiments for Showing the Multiobjective Algorithmic Superiority

#### 4.1.1. Experimental Background

Currently, the heuristic algorithms employed to solve the image color quantization problem have mainly GA, PSO, and DE. Reference [16] indicated that PSO is superior to GA. In [31], DE and PSO show similar performance on image color quantization. However, due to simple operation, litter parameters, and fast convergence, DE is the better choice to use than PSO. These mean that DE is a competitive image color quantization in the heuristic algorithms for image color quantization. Reference [19] proposed a color image quantization algorithm based on self-adaptive hybrid DE (SaDE-CIQ), in which the parameters of DE are automatically adjusted by a self-adaptive mechanic. Then, SaDE-CIQ is compared with *K*-means and the color image quantization algorithm using PSO (PSO-CIQ).  Table 2 shows the smallest and the largest objective values for the three algorithms over 10 runs obtained in [19]. The results show that SaDE-CIQ is an effective color image quantization algorithm, and SaDE-CIQ has better quantization quality than *K*-means and PSO-CIQ. It is naturally to be thought that SaDE-CIQ is the best one of the image color quantization algorithms based on heuristic algorithms.

Reference [28] presented a linear weighting objective function of ${\bar{d}}_{max}$ and *d*_min_ and MSE below:

(10) $$\begin{array}{c} g=w_{1}{\bar{d}}_{max}+w_{2}\left(\;255-d_{min}\;\right)+w_{3}\cdot MSE, \end{array}$$

where *w*_1_, *w*_2_, and *w*_3_ are the user-defined weights of the subobjectives. The linear weighting objective function (10) is the only one, including the three evaluation criteria of MoDE-CIQ, in existing references. So in this section, we will compare MoDE-CIQ, SaDE-CIQ, and SaDE-CIQ3 obtained by replacing the objective function MSE with the linear weighting objective function (10) in SaDE-CIQ.

#### 4.1.2. Experimental Design

MoDE-CIQ, SaDE-CIQ, and SaDE-CIQ3 are implemented to quantize the four test images in Figure 1 into the quantized images with 16 colors. Each algorithm is run 10 times. The parameters of algorithms are set as follows:

 
*K* = 16, NP = 100, *t*_max_ = 200, *Loop* = 5. Mixed probability *p* takes three different values, 0.1, 0.05, and 0.01.   *w*_1_, *w*_2_, and *w*_3_ take the same values as those in [28].

#### 4.1.3. Experimental Results

For MoDE-CIQ, Table 3 reports the best MSE values and the corresponding objective values ${\bar{d}}_{max}$, *d*_min_ in 10 runs. In fact, smaller ${\bar{d}}_{max}$ is better, larger *d*_min_ is better, and smaller MSE is better. As shown in Table 3, the following conclusions are obtained. (i) For Peppers, only MSE is best as *p* = 0.05. ${\bar{d}}_{max}$ and *d*_min_ are best as *p* = 0.01. As *p* = 0.1, ${\bar{d}}_{max}$, *d*_min_, and MSE are all medians, and ${\bar{d}}_{max}$ and MSE are similar to their corresponding best values. (ii) For Baboon, as *p* = 0.1, *d*_min_ and MSE are all best. (iii) For Lena, ${\bar{d}}_{max}$ and MSE are all best as *p* = 0.1. (iv) For Airplane, as *p* = 0.05, ${\bar{d}}_{max}$ is best, *d*_min_ is a median, and MSE is similar to the other two values.

According to the above conclusions, we will take *p* as 0.1 for Peppers, Baboon, and Lena in the following comparing experiments. However, there are few and extremely unequally distributed base colors in Airplane. For preserving main color gradations and richer color levers of original images, ${\bar{d}}_{max}$ should be as small as possible. So we will take *p* as 0.05 for Airplane in the following comparing experiments.

**Figure 3:**
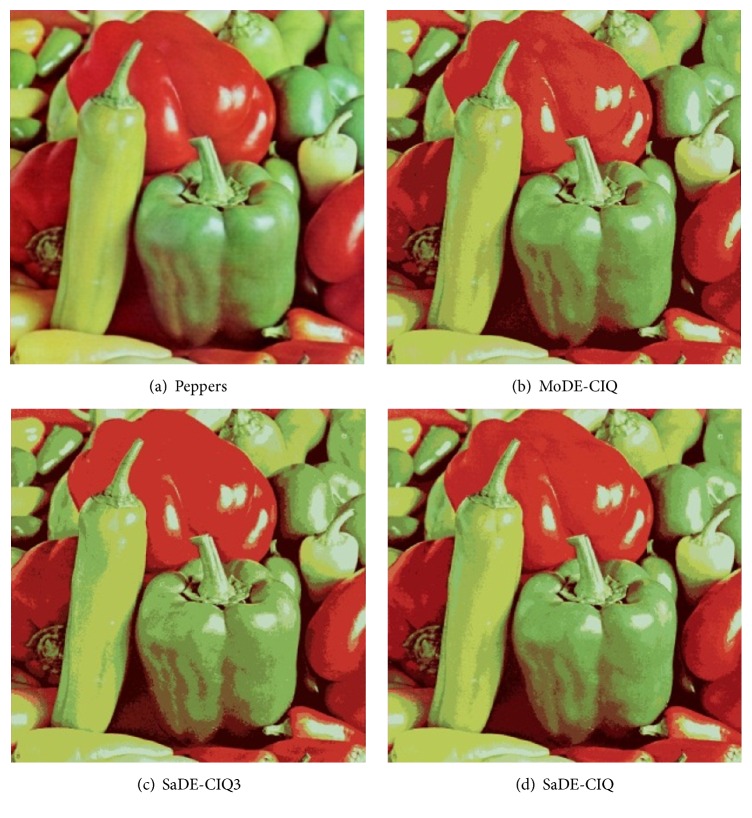
The quantized images of Peppers with 16 colors obtained by three algorithms.

**Figure 4 fig4:**
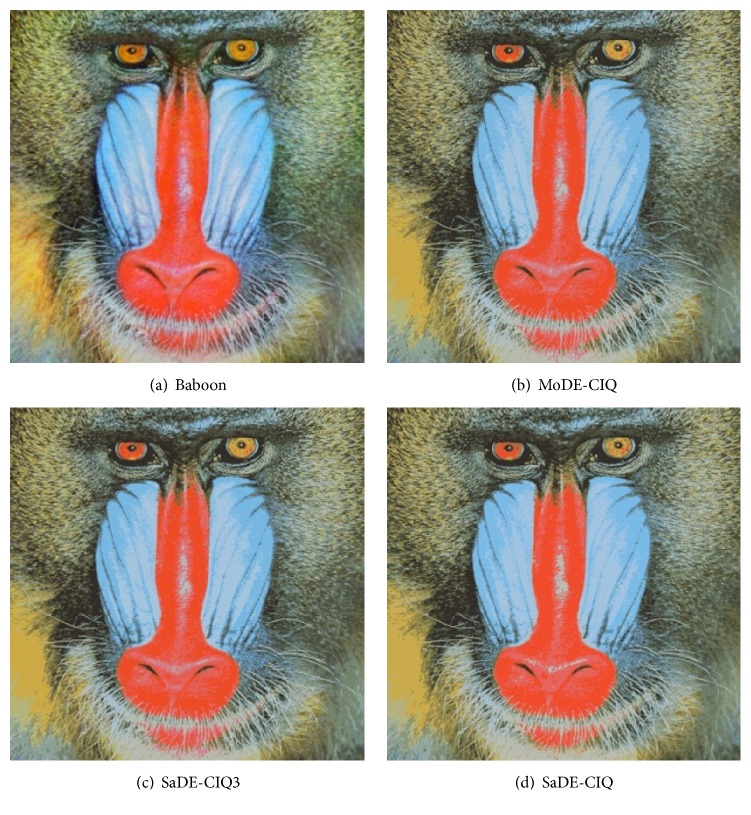
The quantized images of Baboon with 16 colors obtained by three algorithms.

**Figure 5 fig5:**
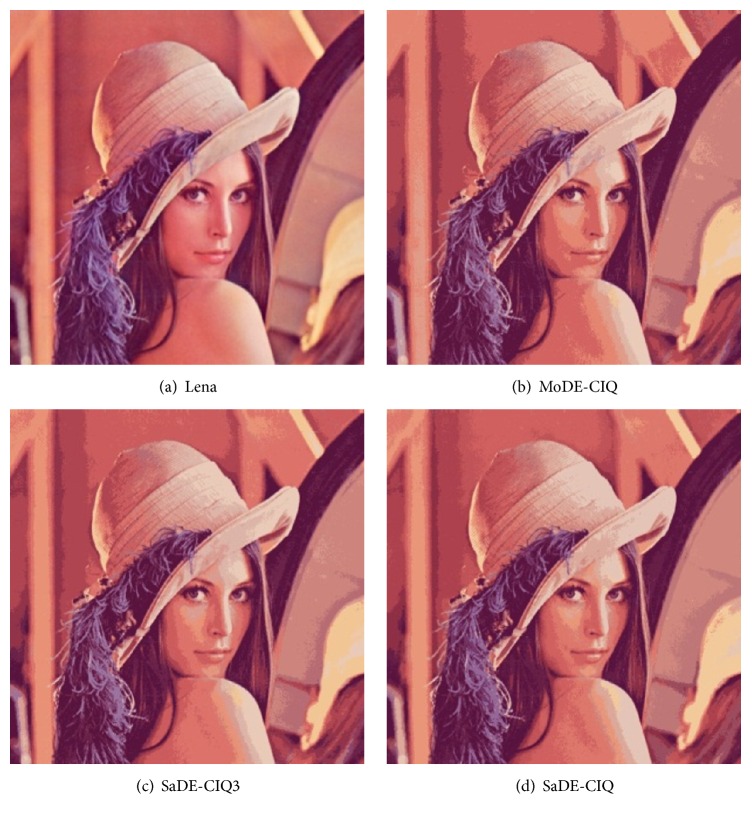
The quantized images of Lena with 16 colors obtained by three algorithms.

**Figure 6:**
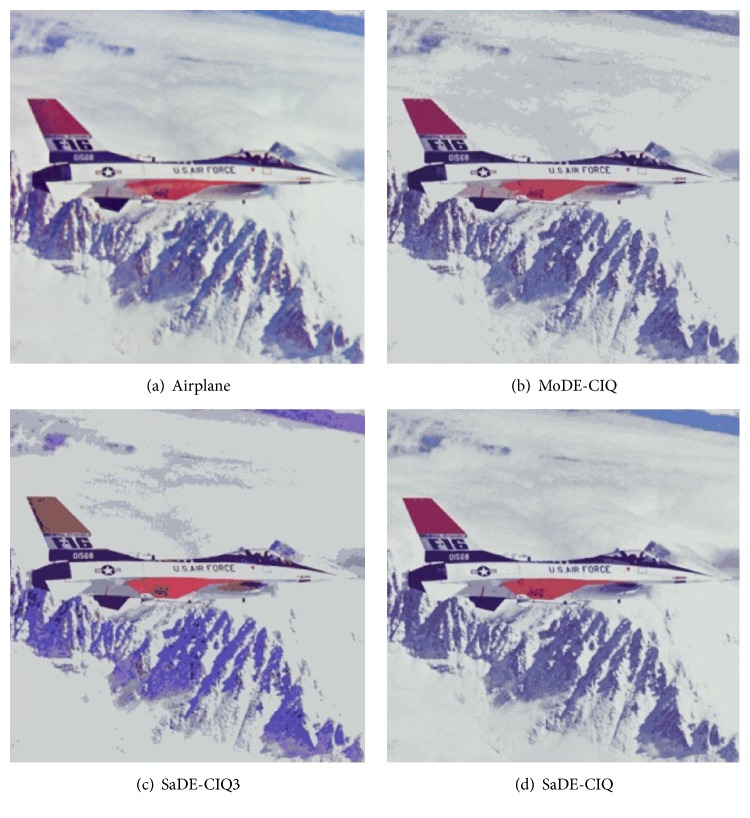
The quantized images of Airplane with 16 colors obtained by three algorithms.

For comparing MoDE-CIQ, SaDE-CIQ, and SaDE-CIQ3, Table 4 reports ${\bar{d}}_{max}$, *d*_min_, and MSE of their quantized images, MSE values of which are the smallest in their 10 runs. SaDE-CIQ aims to minimize its objective MSE, so its values of MSE are surely the best than those of other two algorithms. But the values of ${\bar{d}}_{max}$ and *d*_min_ by MoDE-CIQ are all better than those of SaDE-CIQ. The values of ${\bar{d}}_{max}$ and *d*_min_ obtained by SaDE-CIQ3 for Peppers and Baboon are also better than those of SaDE-CIQ. The values of ${\bar{d}}_{max}$, *d*_min_, and MSE obtained by MoDE-CIQ are better than those of SaDE-CIQ3, except for their similar values of ${\bar{d}}_{max}$, *d*_min_, and MSE for Baboon, and the values of MSE for Lena. Figures 3, 4, 5, and 6 show all quantized images of the four common test images in Figure 1. In Figures 3 [Fig fig4] [Fig fig5]–6, all subfigures (a) are the original test images. Subfigures (b), subfigures (c), and subfigures (d) are the quantized images separately obtained by MoDE-CIQ, SaDE-CIQ3, and SaDE-CIQ. The visual effects of the quantized images are compared as follows. (i) For Peppers (shown in Figure 3), there are contrasting and equally distributed main base colors, so the quantized images obtained by three algorithms visually have similar color distortions. The differences in the quantization quality of these quantized images depend on their color gradations of larger regions with similar colors. The quantized images of MoDE-CIQ and SaDE-CIQ have the more rich color levers than the one of the SaDE-CIQ3. (ii) For Baboon (shown in Figure 4), there are also contrasting and equally distributed main base colors, but there are little larger regions with similar colors. So the quantized images of three methods have similar effects. (iii) For Lena (shown in Figure 5), there are many shaded regions in it. So differences in the quantization quality of the corresponding quantized images depend on the transition from shaded regions to highlights. MoDE-CIQ obtains the quantized image with more natural transition than SaDE-CIQ and SaDE-CIQ3. (iv) For Airplane (shown in Figure 6), there are extremely unequally distributed base colors. Obviously, the quantized image of SaDE-CIQ3 has the largest color distortion. Although the quantized image of SaDE-CIQ has a little better color distortion than that of the multiobjective algorithm, the former loses some detail colors, such as the cloud in the sky.

According the above results, for the images with contrasting and equally distributed main base colors, the quantization effects of MoDE-CIQ and SaDE-CIQ are similar. But for the images with many shaded regions and extremely unequally distributed base colors, MoDE-CIQ could make the colors more natural and preserve more detail colors. In SaDE-CIQ3, the weighted factors in (10) affect its quantization quality. Thus, we can think MoDE-CIQ is superior to the other two algorithms.

### 4.2. Experiments for Showing the Advantage of the Multiobjective Model

**Figure 7:**
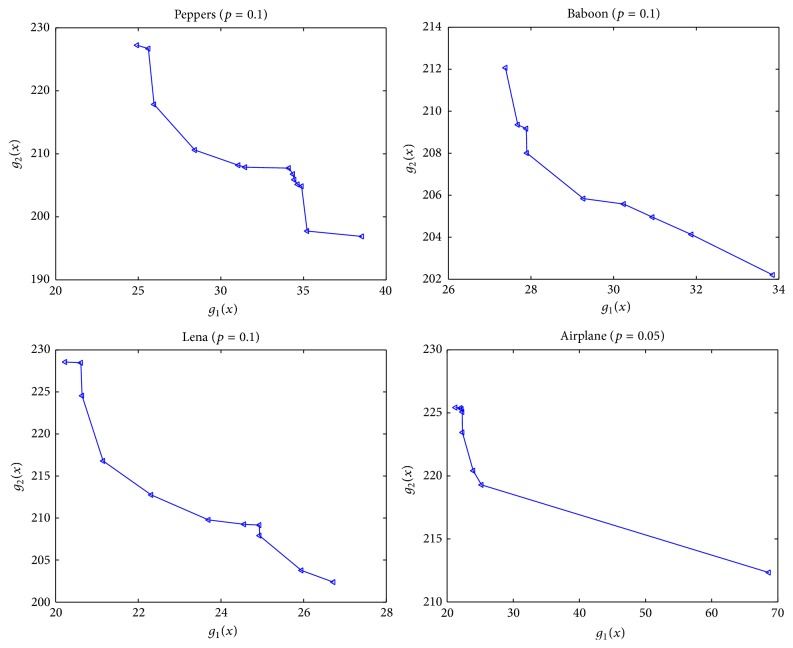
Pareto front of MoDE-CIQ.

As the statement on Step 4 of MoDE-CIQ, we can obtain an approximative Pareto solution set. This is an advantage comparing to all single-objective algorithms. The above experiments reserved the approximative Pareto-optimal solutions of all four images. The solution sets corresponding to Peppers, Baboon, Lena, and Airplane, respectively, include 13 solutions (shown in Table 5), 9 solutions (in Table 6), 11 solutions (in Table 7), and 8 solutions (in Table 8). For comparing these optimal solutions, their corresponding MSE values are listed.  Figure 7 shows the Pareto front of these Pareto-optimal solutions. These optimization solutions present some quantized images with different effects. Users can select the suitable quantized image according to their requirements for the color gradations and details.

By the experimental results of the above two parts, MoDE-CIQ is a competitive algorithm for image color quantization.

All the above algorithms were implemented in Visual C++ and the experiments were conducted on a computer with Intel® Xeon® CPU E3-1230 v3 @ 3.30 GHZ and 8 GB RAM.

## 5. Conclusions

This paper established a multiobjective image color quantization model, in which intracluster distance ${\bar{d}}_{max}$ and intercluster separation *d*_min_ are selected as its objective functions. A multiobjective image color quantization algorithm based on self-adaptive hybrid DE (MoDE-CIQ) was proposed to solve this model. MoDE-CIQ emerges the ideas of SaDE-CIQ [19] and a multipopulation DE algorithm VEDE [30], and applies MSE to determine the optimal solution. The multiobjective model and the proposed algorithm present a strategy to obtain a quantized image which holds the smallest color distortion among those images with a balance between the optimal color gradation and the optimal color details. The experimental results indicated that the multiobjective model and MoDE-CIQ are effective and competitive for image color quantization problems.

## Figures and Tables

**Pseudocode 1:**
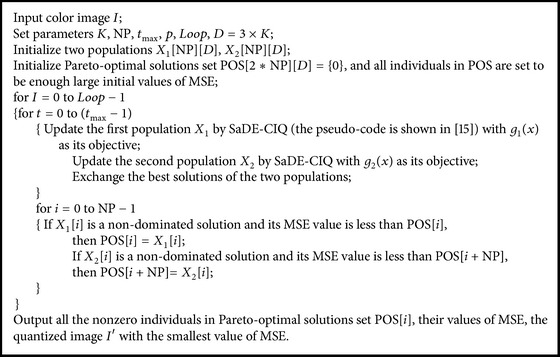
The pseudocode of MoDE-CIQ.

**Table 1:** The results of 10 runs for SaDE-CIQ1 (*p* = 0.1).

Test image		Test serial number									
		1	2	3	4	5	6	7	8	9	10
Peppers	${\bar{d}}_{max}$	27.8885	26.9858	26.0681	28.1934	25.7472	32.8054	26.8729	32.0317	25.5597	28.0979
	*d*_min_	41.5508	29.3541	25.0297	37.4886	40.54	33.6902	35.1127	36.4135	38.3825	36.1761
	MSE	26.8221	26.2667	25.1661	26.8304	25.5318	31.5104	24.1938	29.6623	23.7019	27.8777

Baboon	${\bar{d}}_{max}$	26.6224	27.6260	28.3537	26.8452	26.6689	30.5386	27.6907	28.4376	26.8122	28.7434
	*d*_min_	36.2255	32.8375	34.9105	24.7064	36.5621	30.9652	26.8745	25.9984	33.4011	38.2255
	MSE	20.3766	20.8404	20.1511	19.6093	20.9290	19.4481	21.6021	20.2362	19.4163	20.5033

Lena	${\bar{d}}_{max}$	27.2745	34.0579	28.5068	26.6540	26.6780	37.2558	12.6332	34.9201	28.0219	33.1166
	*d*_min_	21.9009	36.3725	33.3204	37.1832	37.1524	26.1205	37.3509	28.9622	24.5176	29.3508
	MSE	8.3868	15.5077	28.0535	5.6724	15.9792	40.6224	9.5826	38.4419	17.6261	13.4949

Airplane	${\bar{d}}_{max}$	21.9009	36.3725	33.3204	37.1832	37.1524	26.1205	37.3509	28.9622	24.5176	29.3508
	*d*_min_	8.3868	15.5077	28.0535	5.6724	15.9792	40.6224	9.5826	38.4419	17.6261	13.4949
	MSE	15.5626	25.9173	26.8238	22.2865	26.5673	20.2143	29.551	25.9917	21.0685	24.9274

**Table 2:** The MSE values resulting from SaDE-CIQ, *K*-means, and PSO-CIQ.

Alg.	Peppers		Baboon		Lena		Airplane	
	Min	Max	Min	Max	Min	Max	Min	Max
SaDE-CIQ	17.4682	18.7266	22.7496	23.3382	12.9709	13.8055	8.2482	8.9740
*K*-means	18.1086	21.2676	22.9532	24.9563	15.6401	19.1314	9.1141	10.4430
PSO-CIQ	36.3436	40.9532	35.8892	41.9940	29.6644	34.5867	21.3540	24.3200

**Table 3:** The best MSE values and the corresponding objective values of MoDE-CIQ.

Image	*p* values	${\bar{d}}_{max}$	*d*_min_	MSE
Peppers	0.1	25.6127	28.2967	19.1029
	0.05	28.2967	31.9070	18.8444
	0.01	24.8917	38.4062	19.5632

Baboon	0.1	27.8841	45.8284	22.9602
	0.05	27.8083	45.5262	22.9887
	0.01	27.9030	44.7175	22.9654

Lena	0.1	20.2311	26.4388	14.2847
	0.05	20.2849	32.0907	15.6655
	0.01	21.1913	32.9181	15.5229

Airplane	0.1	22.0570	24.1028	10.7517
	0.05	22.0105	29.6160	11.2520
	0.01	20.9759	26.9999	10.9591

**Table 4:** ${\bar{d}}_{max}$, *d*_min_, and MSE of the quantized images with 16 colors by three algorithms.

Image	*p* values	Algorithm	${\bar{d}}_{max}$	*d*_min_	MSE
Peppers	0.1	MoDE-CIQ	25.6127	28.2967	19.1029
		SaDE-CIQ3	34.2489	45.8673	20.3563
		SaDE-CIQ	37.2450	22.2473	17.4577

Baboon	0.1	MoDE-CIQ	27.8841	45.8284	22.9602
		SaDE-CIQ3	27.8122	45.8426	22.9592
		SaDE-CIQ	28.1805	36.4773	22.7644

Lena	0.1	MoDE-CIQ	20.2311	26.4388	14.2847
		SaDE-CIQ3	22.8824	27.5461	13.5264
		SaDE-CIQ	22.2973	19.0143	12.9641

Airplane	0.05	MoDE-CIQ	22.0105	29.6160	11.2520
		SaDE-CIQ3	113.2050	34.8630	17.4217
		SaDE-CIQ	23.7529	8.2540	8.0544

**Table 5:** Pareto-optimal solutions for Peppers.

Order	*g*_1_(*x*)	*g*_2_(*x*)	MSE
1	24.9238	227.2425	19.5660
2	38.5345	196.8913	25.4623
3	31.0556	208.1886	24.1790
4	34.6405	205.1429	22.2026
5	25.6127	226.7033	19.1029
6	35.2191	197.7366	23.0621
7	31.4675	207.8684	23.3818
8	34.4429	205.8758	22.2859
9	34.8841	204.8451	26.031
10	34.1102	207.7311	23.1158
11	25.9563	217.8530	20.4536
12	28.4238	210.6036	22.2533
13	34.3636	206.7747	21.8853

**Table 6:** Pareto-optimal solutions for Baboon.

Order	*g*_1_(*x*)	*g*_2_(*x*)	MSE
1	27.3819	212.0693	23.1123
2	27.8841	209.1716	22.9602
3	31.8821	204.127	24.7008
4	29.2681	205.8375	24.4271
5	30.9412	204.9553	24.6433
6	33.8514	202.2041	25.7050
7	30.2455	205.5812	24.5084
8	27.8998	208.0066	227.341
9	27.6801	209.3535	227.341

**Table 7:** Pareto-optimal solutions for Lena.

Order	*g*_1_(*x*)	*g*_2_(*x*)	MSE
1	24.9313	207.9088	19.8533
2	20.2311	228.5612	14.2847
3	20.6109	228.4630	15.1880
4	26.7185	202.3789	20.9155
5	25.9452	203.7679	20.8445
6	24.5586	209.2558	18.9967
7	23.6997	209.7721	19.8327
8	22.3126	212.7511	20.1288
9	21.1493	216.7855	17.3328
10	24.9279	209.1586	233.6600
11	20.6396	224.5480	233.6600

**Table 8:** Pareto-optimal solutions for Airplane.

Order	*g*_1_(*x*)	*g*_2_(*x*)	MSE
1	23.9419	220.4130	12.4973
2	21.2536	225.4198	11.4192
3	22.3128	223.4460	11.5732
4	22.0105	225.3840	11.2520
5	22.2876	225.0752	11.4864
6	25.2011	219.2880	13.5785
7	68.6311	212.3398	316.6950
8	22.1871	225.3009	316.6950
